# Diabetes is associated with greater leg pain and worse patient-reported outcomes at 1 year after lumbar spine surgery

**DOI:** 10.1038/s41598-021-87615-y

**Published:** 2021-04-14

**Authors:** Kosei Nagata, Hideki Nakamoto, Masahiko Sumitani, So Kato, Yuichi Yoshida, Naohiro Kawamura, Keiichiro Tozawa, Yujiro Takeshita, Hiroyuki Nakarai, Akiro Higashikawa, Masaaki Iizuka, Takashi Ono, Masayoshi Fukushima, Katsuyuki Sasaki, Rentaro Okazaki, Yusuke Ito, Nobuhiro Hara, Toru Doi, Yuki Taniguchi, Yoshitaka Matsubayashi, Sakae Tanaka, Yasushi Oshima

**Affiliations:** 1 Department of Orthopaedic Surgery and Spinal Surgery, The University of Tokyo, 7-3-1 Hongo, Bunkyo-ku, Tokyo, 113-8655 Japan; 2 Department of Pain and Palliative Medicine, The University of Tokyo, Tokyo, Japan; 3 Department of Spine and Orthopedic Surgery, Japanese Red Cross Medical Center, Tokyo, Japan; 4 Department of Orthopedic Surgery, Yokohama Rosai Hospital, Yokohama, Kanagawa Japan; 5 Department of Orthopedic Surgery, Kanto Rosai Hospital, Kawasaki, Kanagawa Japan; 6 Department of Spinal Surgery, Japan Community Health-Care Organization Tokyo Shinjuku Medical Center, Tokyo, Japan; 7 Spine Center, Toranomon Hospital, Tokyo, Japan; 8 Department of Orthopedic Surgery, Japanese Red Cross Saitama Hospital, Saitama, Japan; 9 Department of Orthopedic Surgery, Japanese Red Cross Musashino Hospital, Tokyo, Japan

**Keywords:** Medical research, Neurology, Signs and symptoms

## Abstract

Although patients with diabetes reportedly have more back pain and worse patient-reported outcomes than those without diabetes after lumbar spine surgery, the impact of diabetes on postoperative recovery in pain or numbness in other regions is not well characterized. In this study, the authors aimed to elucidate the impact of diabetes on postoperative recovery in pain/numbness in four areas (back, buttock, leg, and sole) after lumbar spine surgery. The authors retrospectively reviewed 993 patients (152 with diabetes and 841 without) who underwent decompression and/or fixation within three levels of the lumbar spine at eight hospitals during April 2017–June 2018. Preoperative Numerical Rating Scale (NRS) scores in all four areas, Oswestry Disability Index (ODI), and Euro quality of life 5-dimension (EQ-5D) were comparable between the groups. The diabetic group showed worse ODI/EQ-5D and greater NRS scores for leg pain 1 year after surgery than the non-diabetic group. Although other postoperative NRS scores tended to be higher in the diabetic group, the between-group differences were not significant. Diabetic neuropathy caused by microvascular changes may induce irreversible nerve damage especially in leg area. Providers can use this information when counseling patients with diabetes about the expected outcomes of spine surgery.

## Introduction

Diabetic neuropathy can cause chronic peripheral pain due to microvascular changes and irreversible nerve damage^1–5^. The reported prevalence of painful diabetic peripheral neuropathy shows wide variability (range, 3–65%) owing to differences in study design, diagnostic criteria, and sampling methods^1–9^. In addition, diabetic neuropathy can profoundly impair the quality of life^1,10^. Compromised vascularity and secondary peripheral neuropathy may affect the recovery of nerve roots after surgical decompression^11^. Therefore, patients with coexisting diabetes and lumbar spine disease may experience significant limitations in overall function^11^.

Two noteworthy studies investigated the effect of diabetes on outcomes of lumbar spine surgery, especially patient-reported outcomes (PROs) and disability associated with low back pain. A secondary analysis of a large prospective study (SPORT study)^11^ assessed the outcomes of surgery for intervertebral disc herniation, spinal stenosis, and degenerative spondylolisthesis; the results showed that patients without diabetes experienced significantly greater improvement in low back pain-associated disability than patients with diabetes. Another large size prospective study^12^ assessed the outcomes of neck surgery and low back surgery; patients with diabetes showed worse pre- and postoperative PROs, which did not improve to the same extent as that in patients without diabetes. However, these studies did not analyze peripheral pain or numbness; in addition, the latter study assessed patients with both neck pain and low back pain. In another small scale retrospective study^13^, patients with diabetes exhibited a tendency for greater visual analog scale scores for low back pain and leg numbness after lumbar spine surgery. Hence, a larger size study based on prospectively collected data is required to evaluate the effect of diabetes on peripheral pain or numbness in back, buttock, leg, and sole areas after lumbar spine surgery.

The primary purpose of this study was to evaluate whether the presence of diabetes is associated with worse peripheral pain or numbness and worse PROs at 1 year following lumbar spine surgery. The authors hypothesized that outcomes of decompression surgery in patients with diabetes will be worse than those in their non-diabetic counterparts owing to microvascular angiopathy and irreversible nerve damage caused by peripheral neuropathy.

## Materials and methods

### Ethics

A prospective spine surgery registry was started at eight institutions in the greater Tokyo metropolitan area, after obtaining approval from the Clinical Research Support Center of the University of Tokyo Hospital (10335-(3)) and the institutional review boards of all participating hospitals i.e., The University of Tokyo Hospital, Japanese Red Cross Medical Center, Yokohama Rosai Hospital, Kanto Rosai Hospital, Japan Community Health-care Organization Tokyo Shinjuku Medical Center, Toranomon Hospital, Japanese Red Cross Saitama Hospital, and Japanese Red Cross Musashino Hospital. The present study was carried out in accordance with the relevant guidelines and regulations/ethical principles of the Declaration of Helsinki. The authors have obtained informed consent form with opt-out method from patients.

### Patients

The authors evaluated a consecutive cohort of patients with 1-year follow-up from April 2017 and June 2018. Patients in this cohort are divided into nine main disease categories, i.e., degenerative spine disease, intervertebral disc hernia, ossification of posterior longitudinal ligament or yellow ligament, spinal deformity, spinal cord tumor, vertebral tumor, spinal trauma, infectious disease, and others. The authors included only patients with degenerative spine disease and intervertebral disc hernia, as per a previous report^11^. The authors analyzed patients who underwent elective decompression or fixation surgery within three levels of lumbar spine. Fixation included posterior interbody fusion, transforaminal interbody fusion, and posterolateral fusion. The exclusion criteria were (1) age < 20 years; (2) including thoracic level; (3) emergency surgery; (4) revision surgery; (5) surgeries involving osteotomy; and (6) surgical site involving > 3 levels.

Patients with diabetes were identified solely through the medical records (self-reported history of diabetes requiring medication)^12^. Data pertaining to glycosylated hemoglobin (HbA1c) levels during the pre- and postoperative period were collected for patients with diabetes. Patients were divided into diabetic group and non-diabetic group. Data pertaining to demographic characteristics and operative parameters were obtained from the registry. The variables included age, sex, body mass index (BMI), smoking status (current or none), American Society of Anesthesiologists (ASA) classification, operative time, and estimated blood loss, as per the previous reports^12^. Use of interbody fusion or endoscopy was also analyzed. The authors recorded intra- and postoperative complications including nerve root damage, dural tear, deep venous thrombosis, surgical site infection, sepsis, and revision surgery within 30 days after surgery.

### Patient-based outcome measures

The authors used a booklet of questionnaires, including the Japanese version of (1) Numerical Rating Scale (NRS) used in a previous report^14^, (2) Oswestry Disability Index (ODI) for assessment of pain-associated disability^15^, and (3) Euro quality of life 5-dimension (EQ-5D)-3L for assessment of health-related quality of life (QOL)^16,17^. The Japanese versions of ODI^15^ and EQ-5D^16^ have been validated previously. The NRS measures the intensity of pain and numbness over the preceding 4 weeks; the scores range from 0 (no pain at all) to 10 (the worst pain imaginable). To evaluate the effect of lumbar spinal decompression both on back pain and radiculopathy, the authors analyzed six NRS domains: back pain, buttock pain, leg pain, leg numbness, sole pain, and sole numbness. The following “missing” rules were applied in the case of missing data: for ODI and EQ-5D: no missing data were allowed because these scales consist of only one item per domain. As for NRS, because the number of questions was more, one missing response per questionnaire was accepted. We assumed missing–at-random for missing data mechanism and performed no imputations for missing data.

### Statistical analysis

Baseline demographic and clinical characteristics in diabetic group and non-diabetic group were compared using the chi-square test for categorical variables and Student *t*-test for continuous variables. Student *t*-test was used to examine the between-group differences with respect to pre- and postoperative NRS, ODI, and EQ-5D scores. For further evaluation of the difference in each outcome score, the authors calculated the adjusted p values by inverse probability weighting method after calculating propensity scores based on seven variables (age, sex, BMI, smoking status, ASA class, operative time, and estimated blood loss) as per a previous report^12^.

In addition to comparing the mean values of NRS, the authors tried to assess the effect of diabetes on the relief in pain or numbness. A 50% reduction was shown to be substantially important for patients treated for pain^18,19^; therefore, the authors defined pain or numbness relief as decrease in NRS score by 50%. Patients who did not have preoperative pain or numbness (preoperative NRS = 0) were judged to have achieved pain or numbness relief if there was no pain 1 year after surgery (postoperative NRS = 0). The rates of achievement of pain or numbness relief were analyzed by chi-square test.

The sample size for this study was calculated based on multiple linear regressions controlling for eight independent variables (diabetes plus seven variables used for propensity score), which required ten cases per variable. Estimating 10–20% of diabetes prevalence as per previous prospectively collected database^1,20^, a total sample size of at least 800 patients was required. The level of significance was set at p < 0.05. All data analyses were performed using SPSS version 21.0 statistical software (SPSS, Inc., Chicago, IL, USA).

## Results

**Figure 1:**
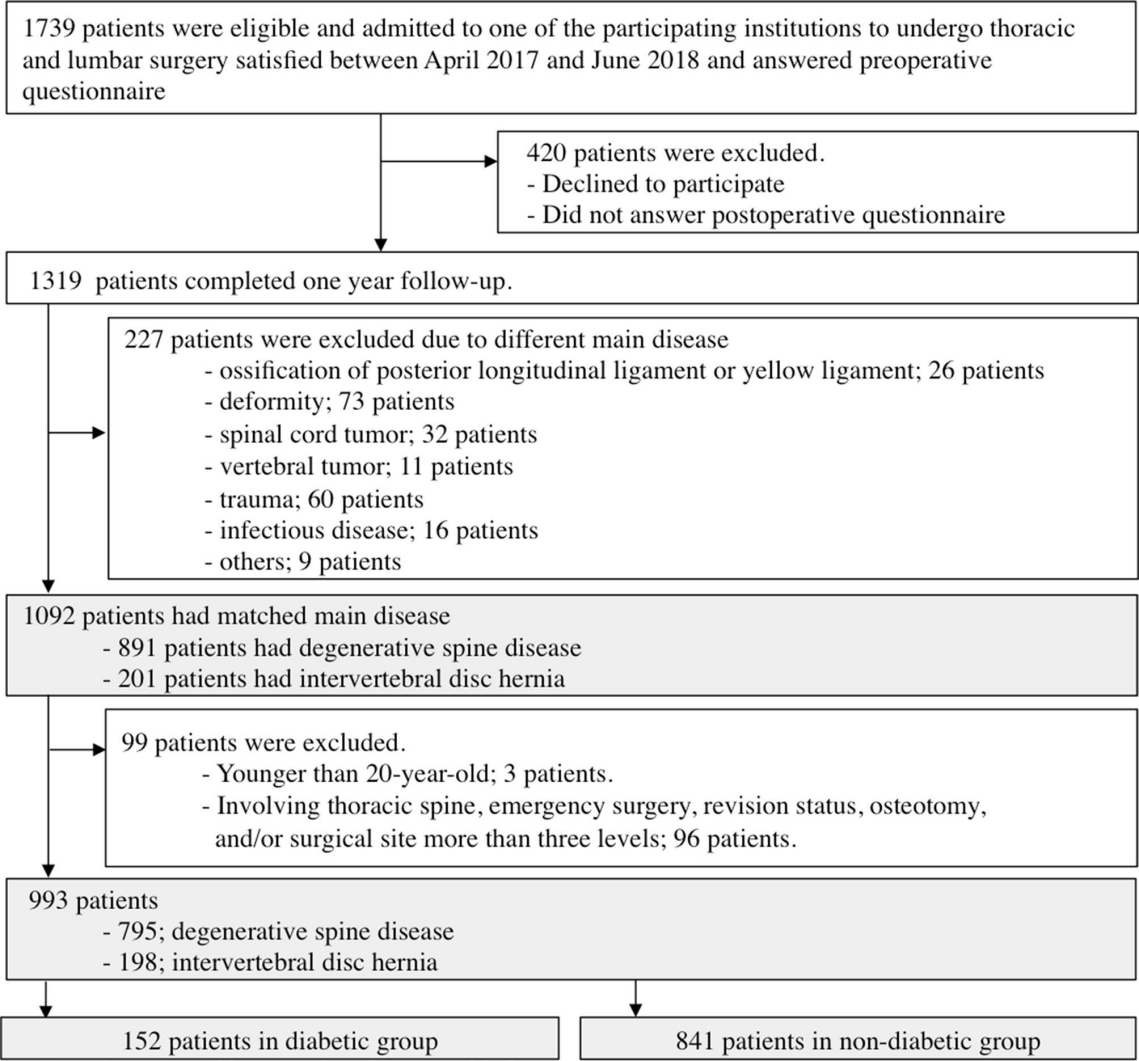
Flowchart showing the selection of participants.

Out of 993 patients who qualified the inclusion and exclusion criteria, 152 patients were in the diabetic group and 841 were in the non-diabetic group (Fig. 1). The diabetic group was significantly older (mean age: 71 vs 66 years, P < 0.001), included more male patients (66% vs 55%, P < 0.001), had higher BMI (26 vs 24 kg/m^2^, P < 0.001), and included a higher percentage of ASA class ≥ 3 (26% vs 7.4%, P < 0.001) than the non-diabetic group. There were no significant between-group differences with respect to smoking status, disease category, or surgical invasiveness (including operative time, estimated blood loss, interbody fusion, and endoscopy use) (Table 1).

**Table 1:** Demographic and patient characteristics (N = 993).

	Diabetic group (N = 152)	Non-diabetic group (N = 841)	P value
Age^a^ (years)	70.7 ± 10.1	66.3 ± 13.9	< 0.001
Male^b^ (%)	65.8	55.1	0.014
BMI^a^ (kg/m^2^)	25.5 ± 4.3	24.0 ± 3.8	< 0.001
Current smoker^b^ (%)	9.2	7.5	0.260
ASA classification ≥ 3^b^ (%)	25.7	7.4	< 0.001
Hernia^b^ (%)	15.8	20.7	0.164
Degenerative^b^ (%)	84.2	79.3	0.164
Operative time^a^ (min)	155.3 ± 90.9	156.7 ± 86.6	0.855
Estimated blood loss^a^ (ml)	206.4 ± 300.0	222 ± 351.7	0.618
Interbody fusion^b^ (%)	32.9	35.3	0.565
Endoscopy use^b^ (%)	23.0	20.3	0.451
Mean preoperative HbA1c^b^ (%)	6.9	−	NA
Insulin user^b^ (%)	13.8	−	NA

BMI, body mass index; ASA, American Society of Anesthesiologists.

^a^The values are given as the mean and standard deviation.

^b^The values are given as the percentage in each group.

The incidences of intra- and postoperative complications were comparable in the two groups (Table 2).

**Table 2:** Comparison of intra- and postoperative complications for patients with diabetes and those without diabetes.

	Diabetic group (N = 152)—number (%)	Non-diabetic group (N = 841)—number (%)	P value
Nerve root damage	0 (0)	2 (0.2)	0.703
Dural tear	2 (1.3)	9 (1.1)	0.877
Deep venous thrombosis	0 (0)	1 (0.1)	0.336
Surgical site infection	1 (0.7)	7(0.8)	0.786
Sepsis	0 (0)	2 (0.2)	0.703
Revision surgery	1 (0.7)	9 (1.1)	0.475

The absolute changes in clinical outcomes are shown in Table 3. No significant difference was observed in any preoperative NRS. Only leg pain was significantly different between the diabetic and non-diabetic groups (3.03 vs 2.45, P = 0.021). The inverse probability weighting method supported this result (adjusted P = 0.013). Although other NRS scores tended to be higher in the diabetic group, the between-group difference was not significant before adjusting (P < 0.05). There was a significant difference in NRS scores for back pain by inverse probability weighting method (3.38 vs 2.95, adjusted P = 0.046). There were no significant between-group differences in preoperative ODI and EQ-5D scores. Patients in the diabetic group showed worse postoperative ODI and EQ-5D scores than those in the non-diabetic group, with or without adjusting (P < 0.05 for both).

**Table 3:** Comparison of preoperative and postoperative scores for patients with diabetes and those without diabetes.

Outcome	Preoperative scores			Postoperative scores (at 1 year)			
	Diabetic group	Non-diabetic group	P value	Diabetic group	Non-diabetic group	P value	adjusted p value
NRS back pain	6.12 ± 3.10	5.64 ± 3.14	0.082	3.38 ± 2.96	2.95 ± 2.70	0.077	0.046
NRS buttock pain	4.91 ± 3.50	5.25 ± 3.44	0.263	1.63 ± 2.49	1.55 ± 2.36	0.719	0.571
NRS Leg pain	6.24 ± 3.13	6.27 ± 3.17	0.902	3.03 ± 3.01	2.45 ± 2.78	0.021	0.013
NRS leg numbness	5.63 ± 3.40	5.55 ± 3.32	0.276	2.61 ± 2.92	2.44 ± 2.80	0.488	0.320
NRS sole pain	2.43 ± 3.12	2.15 ± 2.99	0.289	1.49 ± 2.57	1.30 ± 2.31	0.366	0.455
NRS sole numbness	3.78 ± 3.58	3.39 ± 3.47	0.207	2.26 ± 2.99	2.10 ± 2.83	0.507	0.463
ODI	48.6 ± 20.3	47.4 ± 18.8	0.466	24.6 ± 19.6	19.8 ± 17.6	0.003	0.003
EQ-5D	0.50 ± 0.19	0.51 ± 0.18	0.334	0.70 ± 0.18	0.74 ± 0.19	0.005	0.004

NRS, Numerical Rating Scale; ODI, Oswestry Disability Index; EQ-5D, Euro quality of life-5 dimension.

The P values were examined by Student’s t-test. The adjusted P values were calculated by the inverse probability weighted logistic regression model, setting the propensity scores based on patients’ age, sex, body mass index, smoking status, American Society of Anesthesiologists classification, operative time, and estimated blood loss.

Table 4 shows the percentage of patients who achieved 50% decrease in postoperative NRS scores in the two groups. A significant between-group difference was observed only with respect to NRS score for leg pain (59.7% vs 68.5%, P = 0.037), although the between- group difference turned out to be not significant in the analyses excluding patients with preoperative NRS leg pain score 0 at baseline (62.9 vs 70.1%, P = 0.099).

**Table 4:** Comparison of rates (%) of achievement of 50% relief in pain/numbness between the two groups.

	Total data			Excluding preoperative NRS = 0 of each score		
	Diabetic group	Non-diabetic group	P value	Diabetic group	Non-diabetic group	P value
NRS back pain	54.1	56.1	0.644	52.2	57.7	0.230
NRS buttock pain	75.2	77.2	0.584	73.6	78.5	0.231
NRS leg pain	59.7	68.5	0.037	62.9	70.1	0.099
NRS leg numbness	63.3	65.8	0.563	63.3	66.9	0.729
NRS planter pain	73.7	72.7	0.792	68.1	70.1	0.729
NRS planter numbness	64.7	64.4	0.953	57.7	59.6	0.738

The values are given as the percentage in each group. Each P value was calculated by chi-square test.

CI, confidence interval; NRS, Numerical Rating Scale.

## Discussion

To the best of our knowledge, this is the largest study that evaluated the effect of diabetes on outcomes of elective lumbar spine surgery using multiple NRS and PROs. In the present study, diabetes was associated with greater postoperative leg pain and worse ODI and EQ-5D 1 year after lumbar spine surgery. Our results pertaining to PRO are consistent and those pertaining to back pain are partially consistent with those of previous studies performed in the US^11,12^.

Our findings pertaining to NRS scores for back pain are partially inconsistent with previous reports^11,12^. They suggested that the lesser improvement in back pain in patients with diabetes was likely attributable to degenerative spine. In the present study, NRS score for back pain was not significantly different before adjusting for other demographic and surgical factors. Our cohort was older by more than 10 years than their cohorts^11,12^. Degenerative lumbar diseases are also referred to as the “aging spine,” which usually causes considerable disability among the elderly^21^. The difference in spine degeneration due to diabetes may have been evened out in our cohort due to the relatively high rates in the elderly. The similar preoperative ODI and EQ-5D scores in patients with or without diabetes in this study can be explained by the similar preoperative NRS scores for back pain. In a previous study, patients with diabetes had worse preoperative disability prior to spine surgery due to back or neck pain^12^; in addition, these patients continued to experience worse PROs at 1 year following surgery compared with patients without diabetes^12^. Although this study did not include image analyses, diabetes is a risk factor of high nonunion rate for fusion surgery^11^. This hypothesis supported that the preoperative PROs and NRS scores were comparable between the two groups despite postoperative back pain being worse in the diabetic group. Because the previous report^12^ showed that surgical type was not associated with PRO at 1 year after surgery, we did not include fusion or nonfusion as a covariate. Additionally, they reported that posterior fusion might be associated with ODI and not with EQ-5D or NRS at 2-year follow-up^12^. Further prospective studies are needed with larger sample sizes in each surgical type, ideally including image analyses data.

The authors observed worse postoperative PROs in the diabetic group, similar to that in previous studies^11,12^. Leg pain was shown to be associated with PROs in patients with lumbar spine pathology^22–24^. The between-group difference in postoperative ODI and EQ-5D were approximately 5 and 0.04, respectively. Such a difference was important, but slight and may not be detectable in clinical scenarios because these figures were below the minimum clinically important differences, which are the threshold levels (ODI = 13^25^ and EQ-5D = 0.19^26^) used to measure the effect of clinical treatments. Importantly, patients with diabetes showed an approximately 24-point improvement in ODI scores and a 0.2-point improvement in EQ-5D scores. Taken together, surgical intervention for degenerative disease and disc herniation is an appropriate treatment plan for patients with diabetes, resulting in clinically important differences from the baseline to 1-year follow-up, although they showed significantly greater leg pain and poorer PROs compared with patients without diabetes. Poor improvement in leg pain in the diabetic group may have contributed to worse postoperative PROs in our relatively old cohort. Leg strength and proprioception are associated with physical function; poor leg strength and proprioception may predispose patients with diabetes to greater risk of falls and slower walking speed^27^.

Diabetic microvascular changes can induce variable degrees of peripheral neuropathy and irreversible nerve damage^11,28^. Our data revealed that diabetic neuropathy had a minor-to-moderate effect on the improvement in NRS score for leg pain; the difference between the diabetic and non-diabetic groups in this respect was approximately 0.6. In a study by Takahashi et al*.*^13^ the final leg pain in patients with diabetes was greater than that in their non-diabetic counterparts (difference of 5 mm on visual analog scale); however, the difference was not statistically significant. In light of these findings, the estimated negative effect of diabetes on NRS leg pain score is in the range of 0.5–0.6. The present study indicated that the rate in achievement of 50% relief in pain/numbness was associated with diabetes. Although the p value for leg pain relief was over 0.05 in the analyses excluding patients with 0 pain at baseline, postoperative leg pain was significantly worse in the diabetic group compared to the non-diabetic group in the chi-square test and inverse probability weighting method (P = 0.021, 0.013, respectively), without preoperative differences. These results supported that the effect of diabetes on leg pain relief was not negligible.

Chronic symmetrical length-dependent sensorimotor polyneuropathy is the most common form of diabetic neuropathy^29^. Theoretically, the occurrence of nerve fiber degeneration is proportional to the nerve length and the blood supply. The length from ganglion to leg is longer than that to the buttock area. The difference between the relief of buttock pain and leg pain may be influenced by the length of the nerve. Subjective symptoms of diabetic neuropathy start bilaterally from distal lower limbs^6^, and the effect of diabetic neuropathy on buttock area may be relatively minor compared with leg area.

Contrary to the theory that diabetic neuropathy affects peripheral area^1,5^, our study showed no significant between-group difference with respect to postoperative sole pain and numbness. This can be explained by statistical reasons. Mean pre- and postoperative NRS scores for sole pain and numbness were < 4, which reflects minor pain^30^. It is difficult to demonstrate difference of changes from small baseline values in the 11-point pain intensity NRS system^31^; therefore, the negligible effect of diabetes on postoperative improvement is likely due to the small baseline values. Use of other scoring system (e.g., 101-point scale NRS) may have demonstrated a significant between-group difference in this respect.

Interestingly, no difference in complication rate was observed between the diabetic and the non-diabetic groups in the present study. Surgical site infection is an important cause of revision surgery, and diabetes is known to be one of the risk factors of surgical site infection as shown in a recent meta-analysis^32^. However, the between-group difference was not significant in 5 out of 12 quoted papers^32^. Higher BMI is also an established risk factor of surgical site infection, and the typical cut-off value is 30 or 35^33^. The average BMI of the patients in the present study was lower than the average BMI reported in previous studies in the US^11,12^. Type 2 diabetes can develop in East Asian patients with a lower mean BMI compared with that in those of European descent^34^. Collectively, the effect of diabetes on surgical site infection or revision surgery can differ according to the population due to the differences in BMI. The effect of ASA classification should be mentioned. The present study included 25.7% of participants with an ASA classification ≥ 3 in diabetic group compared to 7.4% in the non-diabetic group. The relatively high-age cohort may mask the effects of unknown comorbidities and optimal medical care provided by the physician, not quantified in this study, can minimize the clinical differences of PRO at the 1-year follow-up between groups. More importantly, the effect of ASA was adjusted by inverse probability weighting method.

The authors believe that this study adds to contemporary knowledge about the effect of diabetes on patients undergoing elective spine surgery. However, some limitations of our study should be considered while interpreting our results. First, the authors were not able to collect detailed information pertaining to diabetes type and baseline glycemic control in patients with diabetes except for the HbA1c values. The type and chronicity of diabetes mellitus and the degree of glycemic control have an impact on neurologic and vascular sequelae of the disease^11^. The degree of vascular calcification and other vascular diseases, including peripheral arterial diseases, were not evaluated in the current study. The authors used inverse probability weighting method calculated by propensity score, and the effect of confounding factors such as ASA classification ≥ 3 can be reduced. Second, the follow-up duration was 1 year, which was shorter than that in the two previous studies^11,12^; however, PROs assessed at 12 months adequately predict the long-term (24-month) outcomes after lumbar spine surgery^35^. Third, we did not approach a response shift in these results. PROs can be influenced by changes in values during study period, and such effects can be evaluated by the “then-test”^36^. Fourth, the authors were not able to analyze detailed information about pain-relief medication because this study was not an interventional study. A previous study only examined the use of opioids^12^, but smaller amount of opioid use was reported in Japan than in the US, especially for acute pain^37^, which may minimize the effect of long-term use of opioid. Another large sample-size study did not evaluate the effect of oral medication between the two groups^11^. Pain control was performed at each outpatient clinic to optimize patients in the two groups, and hence, the confounding factor of the oral medication can be considered negligible for this result.

## Conclusion

Diabetes was associated with poor improvement in leg pain and worse PROs 1 year after spine surgery. Providers can use this information and pay attention to recovery in leg pain as well as back pain when counseling patients with diabetes about the expected outcomes of spine surgery.

## Data Availability

The datasets generated and/or analyzed during the current study are available from the corresponding author on reasonable request.
